# Supplementary material for: Polymorphisms in ARNTL/BMAL1 and CLOCK Are Not Associated with Multiple Sclerosis in Spanish Population
Source: Biology (Basel). 2022 Sep 28;11(10):1417. doi: 10.3390/biology11101417 (PMC9598657; doi:10.3390/biology11101417)
Supplement: Supplementary file 1 [file biology-11-01417-s001.zip › biology-1850614-supplementary.pdf]

Logistic regression and stratified analyses for rs3789327

Logistic regression of the SNP

**Rs3789327**	**OR (95% CI)**	**P**
CC vs. Carrier of T	1.13 (0.89-1.44)	0.30

SNP adjusted by sex, HLA and clinical form

**Rs3789327**	**OR (95% CI)**	**P**
CC vs. Carrier of T	1.06 (0.77-1.47)	0.71
Sex male	1.52 (1.13-2.03)	0.005
Sex female	4.13 (1.06-16.11)	0.041
HLA-DRB1*1501	3.10 (2.23-4.31)	<0.001
Clinical form	--	--

Analysis stratified by sex

***ARNTL* rs3789327**	**FemaleMS**		**FemaleControls**		**p**
	N	%	N	%	
TT	165	*26.4*	87	*24.0*	0.07*
TC	324	*51.9*	175	*48.3*	
CC	135	*21.6*	100	*27.6*	
CC vs Carrier of T	489	*78.4*	262	*72.4*	0.04 (OR=1.38 [1.02-1.86])*
	**Male MS**		**MaleControls**		
TT	78	*25.2*	51	*22.1*	0.92
TC	157	*50.6*	130	*56.3*	
CC	75	*24.2*	50	*21.6*	
CC vs Carrier of T	235	*75.8*	181	*78.4*	0.53

*Statistical comparisons do not standBonferroni correction (p*2)

Analysis stratified by clinical form

***ARNTL*rs3789327**	**RR**		**SP**		**PP**	
	N	%	N	%	N	%
TT	111	*25.4*	23	*27.1*	3	*16.7*
CT	226	*51.7*	43	*50.6*	7	*38.9*
CC	100	*22.9*	19	*22.4*	8	*44.4*

RR vs SP p=0.79**RR vs PP** p=0.07* **SP vs PP** p=0.08*

RR: Relapsing remitting

SP: secondary progressive

PP: primary progressive

*Statistical comparisons do not pass Bonferroni correction (p*2). The size of the PP group is too small to draw solid conclusions

Analysis stratified by HLA DRB*15:01 status

ARNTL rs3789327	**MS**		**Controls**		
**Not HLA-DRB1*15:01**	N	%	N	%	*P*
TT	152	*24.8*	100	*24.6*	0.42
CT	329	*53.6*	204	*50.3*	
CC	133	*21.6*	102	*25.1*	
**HLA-DRB1*15:01 / X**					
TT	82	28.3	14	25.4	0.79
CT	139	47.9	31	56.4	
CC	69	23.8	10	18.2	
**HLA-DRB1*15:01 homozygote**					
TT	7	24.1	0	0	0.61
CT	12	41.4	2	66.7	
CC	10	34.5	1	33.3	

*Statistical comparison does not pass Bonferroni correction (p*2). The control group is small and does not allow drawing solid conclusions.

Logistic regression and stratified analysis for rs6811520

Logistic regression of the SNP

Rs6811520	OR (95% CI)	P
Cc vs. Carrier of T	0.97 (0.72-1.3)	0.82

SNP adjusted by sex, HLA and clinical form

Rs6811520	OR (95% CI)	P
CC vis. Carrier of T	1.09 (0.74-1.63)	0.645
Sex male	1.56 (1.17-2.08)	0.002
Sex female	4.03 (1.03-15.75)	0.045
HLA-DRB1*1501	3.31 (2.40-4.58)	<0.001
Clinical form	--	--

Analysis stratified by sex

CLOCK **rs6811520**	**FemaleMS**		**FemaleControls**		**p**
	N	%	N	%	
TT	265	*41.5*	154	*42.1*	0.83
CT	292	*45.8*	167	*45.6*	
CC	81	*12.7*	45	*12.3*	
	**Male MS**		**MaleControls**		
TT	127	*41.1*	90	*37.3*	0.37
CT	137	*44.3*	112	*46.5*	
CC	45	*14.6*	39	*16.2*	

Analysis stratified by clinical form

CLOCK **rs6811520**	**RR**		**SP**		**PP**	
	N	%	N	%	N	%
TT	179	*41.7*	28	*34.6*	10	*55.5*
CT	199	*46.4*	42	*51.9*	2	*11.2*
CC	51	*11.9*	11	*13.6*	6	*33.3*

RR vs SP p=0.27;**RR vs PP** p=0.64;**SP vs PP** p=0.95

RR: Relapsing remitting

SP: secondary progressive

PP: primary progressive

Analysis stratified by HLA DRB*15:01 status

***CLOCK* rs6811520**	**MS**		**Controls**		
**Not HLA-DRB1*15:01**	N	%	N	%	*p*
TT	244	*39.7*	159	*37.9*	0.26
CT	292	*47.5*	193	*46.1*	
CC	79	*12.8*	67	*16.0*	
**HLA-DRB1*15:01 / X**					
TT	132	*44.9*	31	*55.4*	0.04*
CT	124	*42.2*	23	*41.1*	
CC	38	*12.9*	2	*3.5*	
**HLA-DRB1*15:01 homozygote**					
TT	12	*38.7*	2	*66.7*	0.23
CT	9	*29.0*	1	*33.3*	
CC	10	*32.3*	0	*0*	

*Statistical comparison does not pass Bonferroni correction (p*2). The control group is small and does not allow drawing solid conclusions.
